# How certainty appraisal might improve both body dissatisfaction and body overestimation in anorexia nervosa: a case report

**DOI:** 10.1186/s40337-018-0216-0

**Published:** 2018-10-05

**Authors:** M. Metral, M. Mailliez

**Affiliations:** 1 University Grenoble Alpes, University Savoie Mont Blanc, LIP/PC2S, F-38000 Grenoble, France; 2 University Grenoble Alpes, University Savoie Mont Blanc, CNRS, LPNC, F-38000 Grenoble, France

**Keywords:** Anorexia nervosa, Body overestimation, Body dissatisfaction, Appraisal tendency framework, Cognitive appraisal of (un)certainty

## Abstract

**Background:**

Patients with anorexia nervosa often report a conscious alteration of body image representation, with both body overestimation and body dissatisfaction. Cognitive and behavioural therapy is effective for treating many psychiatric disorders but often fails to treat anorexia nervosa and body image distortions. Although patients are aware of their weight loss, they continue to feel overweight - as if there were a conflict between a previous (maybe already false) body representation and the new one. These distortions are linked to negative emotions focused on the body but which can extend to the self (e.g. disgust and sadness).

**Case Presentation:**

The present case report is the first in which the Appraisal Tendency Framework has been applied to decrease body image distortions in a patient with anorexia nervosa. The Appraisal Tendency Framework is usually used to understand how emotions influence decision making. Here, we report on a 24-year-old woman who suffered from anorexia nervosa and body image distortions, and was treated as an inpatient with conventional cognitive and behavioural therapy for an eating disorder. Body image distortions were assessed before and after the patient completed an adaptation of the Iowa Gambling Task, coupled with the induction of a heuristic processing emotion. We hypothesized that allowing the patient to focus on the emotional cues in the modified Iowa Gambling Task would improve her decisions about her true body shape.

**Conclusion:**

All body image measures were improved after the protocol. Consequently, we suggest that the Appraisal Tendency Framework might be a valuable means of investigating body image issues in eating disorders and anorexia nervosa. Further studies are required to expand and detail these findings.

**Electronic supplementary material:**

The online version of this article (10.1186/s40337-018-0216-0) contains supplementary material, which is available to authorized users.

## Background

Although low self-esteem, depressive symptoms and suicide risk are frequent among patients presenting with anorexia nervosa, most of these symptoms are significantly reduced by treatment (for a review, see [12]). In contrast, the marked body representation distortions associated with anorexia nervosa are not always modified by treatment and thus can lead to relapse [10, 28]. Although patients are aware of their weight loss, they continue to feel overweight - as if their new body schema has not been taken into account or updated [14, 19, 24]. Body representation distortions are currently evaluated in terms of both body overestimation and body dissatisfaction. Some researchers have suggested that cognitive dysfunction in anorexia nervosa may enhance the associated symptoms [15, 20, 23]. There is a growing body of literature data on cognitive impairments and cognitive remediation therapy (CRT) in anorexia nervosa (for a systematic review, see [22]). Four randomised controlled trials demonstrate that CRT has the potential of enhancing the effectiveness of current treatments, reduce attrition and eating disorder psychopathology. We consider that reducing cognitive symptoms in general and decision making about one’s own body in particular may be a potentially valuable means of remediating body representations.

It is well known that patients with eating disorders make disadvantageous decisions in the Iowa Gambling Task (IGT) [7] - just like patients suffering from addiction (e.g. gambling). More precisely, anorexia nervosa is characterized by a tendency to make decisions that have positive short-term outcomes but negative long-term outcomes [6].

Interestingly, this impairment was also observed in participants induced to feel an uncertainty-associated emotion. In contrast, participants induced to feel a certainty-associated emotion made advantageous decisions [1, 5]. According to the Appraisal Tendency Framework (ATF), each emotion induced prior to the decision is defined by a very high or very low score in a set of appraisals (namely pleasantness, anticipated effort, control, responsibility, attentional activity, and *certainty* [26]). More precisely, (un)certainty is one of the most important factors to be appraised in the decision-making process. The appraisal of (un)certainty-associated emotions corresponds to the two ends of a continuum reflecting the degree to which past events are understandable and future events are predictable [26].

The appraisal of (un)certainty modulates the depth of information processing [16, 21]. Emotions induced by the appraisal of certainty lead to more heuristic processing, whereas emotions induced by the appraisal of uncertainty lead to more deliberative processing [29]. Heuristic processing is an automatic, associative type of processing that allows participants to (i) process a large amount of information **(**independently of the cognitive load in working memory) and (ii) learn more rapidly [2, 9, 13, 17, 25]. Due to the automaticity of emotions, heuristic processing allows a person to process emotionally charged information more effectively [18]. In the IGT, participants do have not have any explicit information about the optimal decisions (i.e., the decisions are ambiguous; see [4]). In such a case, heuristic processing is efficient because it takes account of emotional cues associated with previous gains and losses (i.e., feedback; [4]). In contrast, deliberative processing is more associated with in-depth information processing and cognitive processes [2, 8, 13, 17]. Deliberative processing can be limited by its high dependence on the cognitive load in working memory. Moreover, deliberative processing is associated with slow learning [13]. Consequently, the presence of large amounts of information prevents deliberative processing from achieving high levels of performance in sequential decision-making tasks.

Consequently, the primary objective of the present case report was to assess the relevance of using the ATF to decrease body dissatisfaction in anorexia nervosa. Conventionally, patients with anorexia make disadvantageous decisions because they do not take account of long-term feedback and the associated emotional cues (as shown in the IGT). This situation is quite similar to observations of the body image in anorexia. Indeed, patients suffering from anorexia nervosa do not take account of feedback on thinness [11]. Patients make disadvantageous decisions about their body shape because they believe that they are fat, even when they are unhealthily thin. To this end, we modified the IGT so that a patient with anorexia nervosa could learn about an accurate representation of her body. We expected that the induction of a heuristic processing would enable the patient to decreases her body overestimation and dissatisfaction. As mentioned above, heuristic processing allows a person to focus on emotional cues and thus might improve decisions about their true body shape.

## Case presentation

Here, we report on a patient who experienced both body overestimation and body dissatisfaction. In January 2017, a 24-year-old woman was referred to the eating disorders unit at the Hospices de Lyon hospital (Lyon, France) for the treatment of anorexia nervosa that had appeared 12 months previously. A standard diagnostic procedure (based on the DSM-5 criteria for eating disorders) revealed anorexia nervosa (F.50). There were no psychiatric or neuroendocrine comorbidities. The patient’s eating disorder was initially characterized by major weight loss over a two-month period. When the CBT-based programme began, the patient’s BMI was 15.94 kg/m^2^. As is often the case with anorexia nervosa, the patient was already dissatisfied with her body (i.e. body dissatisfaction and overestimation). She also failed to accept the changes associated with the weight lost due to the disease, and was still highly dissatisfied with her body, stating “*I feel as if I am obese*”.

Two experimental tasks were used to investigate the patient’s body representation. In the first task, we assessed body size estimation by presenting 23 body shapes four times in random order. Each shape was associated with a BMI standard, ranging from 11 kg/m^2^ to 32 kg/m^2^. In total, 92 different body shape silhouettes were presented. For each silhouette, the patient had to decide whether the depicted body was fatter than, thinner than or equivalent to her own body. The BMI for the stated mean equivalent body shape was then compared with the participant’s actual BMI. The difference between the two values defined the degree of body under- or over-estimation (Table 1). In the second task, we used the same 92 silhouettes (presented over four trials and in random order) to assess body size dissatisfaction. The patient had to decide whether the shape in the photograph was a desirable body shape or not, according to her own standard. The mean BMI for the desirable body shape was then compared with the participant’s perceived BMI. The difference between the two values defined the degree of body dissatisfaction (Table 1).

**Fig. 1:**
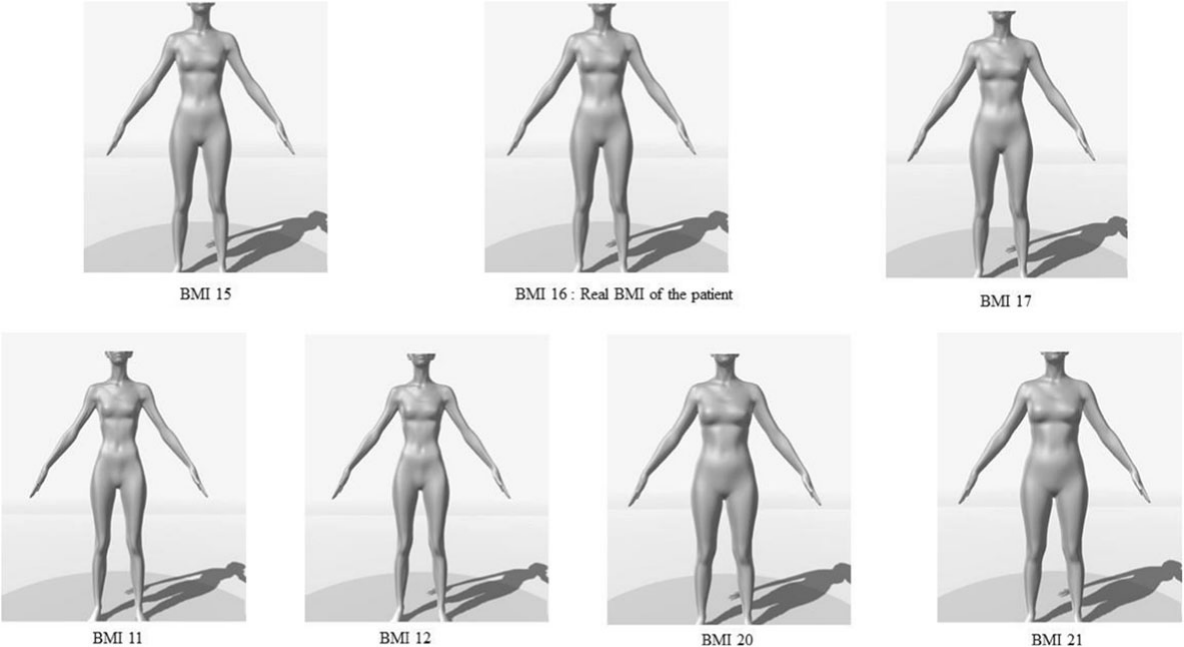
The various body shape items generated as a function of the patient’s true BMI. The patient’s true BMI is 16, so body shapes with a BMI of 17, 16 or 15 are considered to be correct. In contrast, body shapes with a BMI of 11, 12, 20 or 21 are considered to be inaccurate (i.e. BMI -4/−5 and BMI + 4/+ 5)

Lastly, we modified the IGT so that it dealt with body shapes. In the modified version of this sequential decision-making task (referred to henceforth as the Body Iowa Gambling Task Modified; BIGTM), the participant chooses to make financial investments in one of four “body image” companies. In the BIGTM, the four companies (named BUC, JOR, NEP and VAM) replace the four original piles of cards. Based on the patient’s BMI, each company generates a body shape item that may or may not represent the participant’s own body (see Fig. 1). In general, administration of the BIGTM might enable a person to learn about an appropriate representation of his/her own body.

Two companies (i.e. NEP and VAM) offer body shape items that are equivalent or close to the patient’s real BMI (BMI + 1 or BMI – 1, randomized between the two companies; for a true BMI of 16, these items respectively correspond to BMI 17 and BMI 15 in Fig. 1). The other two companies (i.e. BUC and JOR) offer body shape items that are far from the patient’s true BMI (BMI + 4 /+ 5; BMI -4/− 5, randomized between the two companies; for a true BMI of 16, these items respectively correspond to a BMI of 11, 12, 20 and 21 in Fig. 1).

The patient was informed that her goal was to invest money in companies that better represented her body, in order to earn money (at least €3750) without making any strategic errors (i.e. without making disadvantageous decisions). To achieve this goal, the patient has to decide which shares to buy. The patient is told that depending on the behaviour of the stock market, the value of the shares can either decrease or increase.

We used exactly the same structure (i.e., the reinforcement rate) as in the original IGT [3]. Consequently, only the interface differed. As in the original IGT, each choice made by the participant leads to either a profit or a loss and the amount/frequency of loss varied. More precisely, investments in the companies offering body shape items that are equivalent or close to the participant’s BMI (i.e., NEP and VAM) are advantageous decisions in the long run. Investing in an advantageous company led to a gain of €50 or only a loss of €50. In contrast, investments in companies (i.e., BUC and JOR) whose offer body shape items far from the patient’s BMI are disadvantageous in the long term. Deciding to invest in a disadvantageous company led to a gain of €100 or a loss of €250. For ten choices, choosing mostly the disadvantageous company leads to an overall loss of €250, whereas choosing mostly the advantageous company leads to an overall gain of €250. As in the stock market version of the classic IGT, the patient received feedback on the consequences of her decision (i.e. monetary gain or loss) after it had been made. Overall, the patient performed four training trials and 100 test trials.

An impairment threshold was then calculated as the ratio between the total number of advantageous decisions and the total number of disadvantageous decisions. The threshold is a good indicator of the proportion of advantageous decisions. When participants reach this threshold, they make mostly disadvantageous decisions. In contrast, when participants do not reach the threshold, they make mostly advantageous decisions. In the present case, it was also important to check whether or not the impairment threshold was reached; this enabled us to check whether the patient had performed the task well (which was indeed found to be the case).

Before performing the BIGTM, the patient was made to feel a certainty-associated emotion (i.e. in order to induce heuristic processing). As mentioned above, heuristic processing should generate feedback that guides the patient toward the correct body representation. Certainty-associated emotions (i.e., anger) were induced by watching a film clip (i.e. American History X, 1:09 min) that had been tested by Schaefer et al. [27] and in a validation study (see the Additional file 1). Overall, the results of the validation study showed that the film clip induced both anger and the highest level of certainty.

### Overview

We expected that a more accurate body representation (thanks to completion of the BIGTM) would decrease the patient’s body overestimation and body dissatisfaction. To that end, we assessed the patient’s BMI, and invited her to complete the body size estimation task (i.e., statement of the estimated body and the desired body) and to then watch the film clip. Immediately afterwards, the patient completed the BIGTM. Lastly, the patient again completed the body size estimation tasks. The entire procedure lasted about 1 h.

## Results

Several variables (i.e. the estimated BMI and the desired BMI, for calculating body overestimation and body dissatisfaction) had been measured before the emotion associated with the certainty appraisal had been induced by the film clip and then before the BIGTM (see Table 1). We observed both body overestimation (+ 3.69 kg/m^2^) and body dissatisfaction (+ 5.44 kg/m^2^). The patient did not meet the threshold for impairment in the BIGTM (< 10). An analysis of the net score for each block of decisions showed that the patient made advantageous decisions about her body throughout the task. After the BIGTM had been completed, the degree of body overestimation had fallen from + 3.69 kg/m^2^ to + 1.26 kg/m^2^, and the degree of body dissatisfaction had fallen from + 5.44 kg/m^2^ to + 1.60 kg/m^2^.

**Table 1:** The patient’s scores before and after the protocol

BMIs, in kg/m^2^	Pre-test	Post-test	Effect (improvement: +; equivalence: =)
True BMI (A)	15.94	15.94	=
Estimated BMI (B)	19.63	17.2	+
Desired BMI (C)	14.5	15.6	+
Body overestimation (B-A)	3.69	1.26	+
Body dissatisfaction (B-C)	5.44	1.60	+

## Discussion

The objective of the present case study was to assess the value of using the ATF [16] to reduce body image distortion in a patient with anorexia nervosa. More precisely, we sought to determine the extent to which the incidental emotions associated with a type of information processing could reduce body overestimation and body dissatisfaction. Our results show that all body overestimation and dissatisfaction measures had been improved after treatment.

One explanation for the present study may be related to the heuristic processing associated with the incidental emotion. Heuristic processing allows participants to take into account emotional cues associated with feedback (i.e., gain and/or loss; [1, 5]). In our protocol, we hypothesized the heuristic processing guides the patient to select the body shape item that best represents their own body shape. However, the present study had limitations. The first limitation relates to what could be described as a “practice effect”, because the participant performed the body overestimation/dissatisfaction tasks twice in about 1 hour. However, the body shape evaluation by a patient suffering from body overestimation would be unlikely to improve her body image - especially since the experimenter did not provide the patient with any feedback after the first evaluation. We are also quite confident that in the present case, the induction of heuristic processing enables the patient to less focus on short-term consequences and more on long-term consequences. Hence, this induction may be of value for reducing body overestimation in anorexia nervosa. One other possible limitation relates to the fact that the disadvantageous decisions in the BIGTM (e.g., BMI + 4/+ 5 and BMI -4/− 5) were randomized independently of the valence (fatter or thinner body shape). One could imagine that a patient suffering from anorexia nervosa would systematically choose the overestimated body shape (e.g., + 4/+ 5) and avoid the underestimated body shape (e.g., − 4/− 5). In future research, we plan to determine the extent to which the valence associated with the inaccurate body shapes in the BIGTM might influence decision making.

Undoubtedly, our present findings must be considered with caution. We do not claim that this result can be generalized. With a view to evaluating the protocol and its generalizability, we have planned large-scale studies on both patients with anorexia nervosa and healthy individuals. Next, we shall have to determine the long-term, post-BIGTM stability of the decrease in body dissatisfaction observed here. Moreover, further work needs to be carried out to assess the benefit of heuristic feedback processing (i.e., certainty-associated emotion) relative to deliberative feedback processing (i.e., uncertainty-associated emotion), or without any induction of emotion. However, the literature data show that pathological gamblers and patients suffering from anorexia nervosa do not perform better in the IGT when certainty is not induced [7, 30]. Hence, we hypothesize that a patient suffering from anorexia nervosa would perform better, only if the BIGTM is associated with heuristic information processing (and especially heuristic processing of the feedback received during the task).

In contrast to most treatments for anorexia nervosa, the present protocol was not intended to directly improve the patient’s body image. In the context of anorexia nervosa, modulation of the body image is not effective in the long term, and dysfunctional beliefs about the body are often refractory to treatment [10]. The present study paradigm echoes a recent literature review on the use of CRT to reduce the impact of cognitive bias on the symptoms of eating disorders [22]. From a process-based psychopathologic perspective, one of the strengths of the present study is the concept whereby remediation of a specific neurocognitive function or a reduction in cognitive bias (i.e. decision making) may help to reduce body image distortions.

In summary, the present case study suggests that the ATF might be valuable a tool for reducing body overestimation/dissatisfaction in anorexia nervosa. Indeed, the ATF might be a valuable component of new CBT protocols for modulating body overestimation in people with anorexia nervosa.

## Additional file

Additional file 1: The results of the validation study showed that the film clip used in this case report induced both anger and the highest level of certainty. (DOCX 36 kb)

## Abbreviations

ATF: Appraisal Tendency Framework
BIGTM: Body Iowa Gambling Task Modified
CBT: Cognitive Behavioural Therapy
CRT: Cognitive remediation therapy
IGT: Iowa Gambling Task
