# Supplementary material for: How certainty appraisal might improve both body dissatisfaction and body overestimation in anorexia nervosa: a case report
Source: J Eat Disord. 2018 Oct 5;6:29. doi: 10.1186/s40337-018-0216-0 (PMC6172742; doi:10.1186/s40337-018-0216-0)
Supplement: Supplementary file 1 — The results of the validation study showed that the film clip used in this case report induced both anger and the highest level of certainty. (DOCX 36 kb) [file 40337_2018_216_MOESM1_ESM.docx]

How certainty appraisal might improve both body dissatisfaction and body overestimation in anorexia nervosa: a case report

M. Metral ^1^, M. Mailliez ^2^

^1^ Univ. Grenoble Alpes, Univ. Savoie Mont Blanc, CNRS, LPNC, F-38000 Grenoble, France.

^2^ Univ. Grenoble Alpes, Univ. Savoie Mont Blanc, LIP/PC2S, F-38000 Grenoble, France

Correspondence for this article should be addressed to Mailliez Mélody,

UFR LLSH Jacob-Bellecombette, F-73 000 Chambéry cedex, France

Tel.: +33-479-758-390, E-mail : melody.mailliez@univ-smb.fr

**Supplemental Materials**

Validation study

To analyze the decrease in both body dissatisfaction and body overestimation associated with the induction of certainty appraisal, we checked the specificity of the emotions induced by the film clip. In the case report (see the main article), we hypothesized was that certainty appraisal has a key role in the improvement of decisions about the own body. Hence, the objective of the present validation study was to ensure that the film clip induced the emotions that were expected to be associated with the appraisal of certainty.

The aim of emotion induction procedures is to produce the expected emotion in a specific and identifiable way (Maryam Fakhrhosseini, 2017). A meta-analysis has shown that the projection of a film clip is the most efficient means of inducing the expected emotion (Westermann, Spies, Stahl, & Hesse, 1996). The literature data also show that the same emotion can be associated with a high or low level of certainty by using various film clips (Bollon & Bagneux, 2013).

In our case report, we used a film clip that had been tested by Schaefer, Nils, Philippot, & Sanchez (2010). However, these researchers did not measure the degree of certainty associated with the film clip. Since the (un)certainty appraisal associated with incidental emotion triggers a specific type of information processing (heuristic *vs.* deliberative) that guides decisions, it is necessary to check the level of certainty triggered by the film clip.

**Method**

Sixty-three female science or humanities students from the Univ. Savoie Mont Blanc (*M_age_*= 20.49, *SD* = 2.03) were included in this validation study. The participants were randomly assigned to one of two experimental conditions (certainty associated anger: *N* = 32; uncertainty associated anger: *N* = 31). Participation counted toward the completion of a psychology course. Each participant gave her written, informed consent to participation in the study.

**Materials**

Information on the study’s materials and the data analyzed (Supplemental Materials and Case report) can be retrieved from osf.io/a96bf.

**Incidental emotion induction.** We used a film clip tested by Schaefer et al. (2010). For certainty-associated anger, we showed the whole the film clip (*American History X,* 1:09 minutes). In order to manipulate the degree of certainty associated with the film clip and produce uncertainty-associated anger, we deleted the end of the clip (*American History X,* 1:01 minutes).

**Measurements of specific emotions.** To check whether or not the film clips induced the expected incidental emotion, we asked participants to report their emotional state by rating their degree of emotion in 10 emotional categories from the Differential Emotions Scale on a 5-point Likert scale, from 1 (*not at all*) to 5 (*completely*) (Philippot, 1993). Consequently, participants reported their degree of joy (for two items, *feeling amused, joyful, merry* and *warmhearted, gleeful, elated,* which were correlated: *r* = .47), fear (two items, *feeling* *fearful, scared, afraid* and *feeling* *anxious, tense, nervous,* which were correlated: *r* =.58), sadness (for a single item: *feeling sad, downhearted, blue*), anger (for a single item: *feeling angry, irritated, mad*), disgust (for a single item: *feeling disgusted, turned off, repulsed*), disdain (for a single item: *feeling disdainful, scornful, contemptuous*), and being surprised (for a single item: *feeling surprised, amazed, astonished*).

Participants also reported the intensity of their emotional state for a single item on a 7-point Likert scale from 1 (*feeling no emotions*) to 7 (*feeling intense emotions*). Furthermore, participants reported their degree of certainty by answering four items from the Dimensional Ratings Questionnaire on an 11-point Likert scale, from 1 (*not at all*) to 11 (*extremely*), (Cronbach’s α [95% confidence interval (CI) *=* .71 [.57, .81]) (Smith & Ellsworth, 1985; see also Bagneux et al., 2013 for a more detailed presentation)*.*

**Procedure**

To prevent participants from guessing the purpose of the experiment, we told them that they were participating in research on visual and auditory memory (Maryam Fakhrhosseini, 2017). The film clip that induced an incidental emotion was presented as being a visual and auditory memory task. The participants then completed the instruments for measuring specific emotions, which were presented as distractor tasks. In order to continue to mask the study’s purpose and design, we then administered a final questionnaire on general knowledge about the film clip; this was of no interest for the present study. At the end of the entire procedure (which lasted roughly 15 min), the participants were fully debriefed.

**Results**

No outliers were identified using the specific emotion measurement tools. The criteria for normality and heterogeneity of the data were met.

**Measurements of specific emotions.** **Intensity.** Four participants (6.34%) did not report the intensity of their emotional state. Consequently, the data from 57 participants were analyzed.

A one-way analysis of variance (ANOVA) of the intensity of the emotional state failed to shown a significant effect of the emotional condition (*F*(1, 57) < 1,$ns$). The mean (standard deviation) values are given in Table S1.

**Incidental emotion induction.**

***Differences between emotional states.***

As expected, the one-way ANOVA of reported anger did not show a significant effect of the emotional condition, *F*(1, 61) >1, *ns*. The mean (standard deviation) values are given in Table S1.

**Measurements of certainty appraisal.** The one-way ANOVA of certainty revealed a significant effect of the emotional condition (*F*(1, 61) = 4.54, *p* < .05, $\eta_{p}^{2}$.= .07). The mean (standard deviation) values are given in Table S1.

[Insert Table S1 here]

**Discussion**

The aim of the present validation study was to check that the two film clips induced the same emotion but with differing degrees of certainty. As expected, our analysis of the intensity and specificity of the induced emotion failed to show a significant difference between the two film clips; both film clips induced anger.

We also checked whether the film clips induced the expected degree of certainty appraisal. As expected, the participants who watched the film clip leading to certainty-associated anger felt more certain than participants who watched the film clip leading to uncertainty-associated anger did. These results are consistent with the previous report by (Bollon & Bagneux, 2013).

In conclusion, the results of our validation study confirmed our choice of the film clips used to induce a high level of certainty in the case study. The chosen film clips induced the expected, specific emotion with the expected degree of certainty.

**References**

Bagneux, V., Font, H., & Bollon, T. (2013). Incidental emotions associated with uncertainty appraisals impair decisions. *Motivation and Emotion*, *37*(4), 818–827. https://doi.org/10.1007/s11031-013-9346-5

Bollon, T., & Bagneux, V. (2013). Can the uncertainty appraisal associated with emotion cancel the effect of the hunch period in the Iowa Gambling Task? *Cognition & Emotion*, *27*(2), 1–9. https://doi.org/10.1080/02699931.2012.712947

Maryam Fakhrhosseini, S. (2017). *Chapter 10 – Affect/Emotion Induction Methods*. *Emotions and Affect in Human Factors and Human-Computer Interaction*. Elsevier Inc. https://doi.org/10.1016/B978-0-12-801851-4.00010-0

Philippot, P. (1993). Inducing and assessing differentiated emotion-feeling states in the laboratory. *Cognition & Emotion*. https://doi.org/10.1080/02699939308409183

Schaefer, A., Nils, F., Philippot, P., & Sanchez, X. (2010). Assessing the effectiveness of a large database of emotion-eliciting films: A new tool for emotion researchers. *Cognition and Emotion*, *24*(7), 1153–1172. https://doi.org/10.1080/02699930903274322

Smith, C. A., & Ellsworth, P. C. (1985). Patterns of cognitive appraisal in emotion. *Journal of Personality and Social Psychology*, *48*(4), 813–838. https://doi.org/10.1037/0022-3514.48.4.813

Westermann, R., Spies, K., Stahl, G., & Hesse, F. W. (1996). Relative effectiveness and validity of mood induction procedures: A meta-analysis. *European Journal of Social Psychology*, *26*(4), 557–580. https://doi.org/10.1002/(SICI)1099-0992(199607)26:4<557::AID-EJSP769>3.0.CO;2-4

**Tables**

**Table S1.** *Mean (standard deviation) scores for intensity, induced emotion, and degree of certainty as a function of the experimental condition*

Table S1

*Mean (standard deviation) scores for intensity, induced emotion, and degree of certainty as a function of the experimental condition*

|  | Incidental emotion induced | | | |
| --- | --- | --- | --- | --- |
|  | Certainty-associated anger | | Uncertainty-associated anger | |
|  | *Mean* | *SD* | *Mean* | *SD* |
| Intensity score | 4.96 | 1.53 | 5.17 | 1.44 |
| Emotion score |  |  |  |  |
| joy | 1.26 | 0.45 | 1.22^a^ | 0.40 |
| fear | 3.37 | 1.17 | 2.98 | 1.23 |
| sadness | 2.56 | 1.14 | 2.10 | 1.09 |
| anger | 3.47 | 1.24 | 3.34 | 1.29 |
| disgust | 3.93 | 1.11 | 3.41 | 1.48 |
| disdain | 2.43 | 1.32 | 1.75 | 1.33 |
| surprised | 2.68 | 1.40 | 2.44 | 1.42 |
| Degree of certainty | 6.92 | 2.04 | 5.75 | 2.22 |
